# Combination of Immune Checkpoint Inhibitors and Liver-Specific Therapies in Liver-Metastatic Uveal Melanoma: Can We Thus Overcome Its High Resistance?

**DOI:** 10.3390/cancers13246390

**Published:** 2021-12-20

**Authors:** Chiara L. Blomen, Julian Kött, Tabea I. Hartung, Leopold K. Torster, Christoffer Gebhardt

**Affiliations:** Department of Dermatology and Venereology, University Medical Center Hamburg-Eppendorf (UKE), 20246 Hamburg, Germany; chiara.blomen@web.de (C.L.B.); j.koett@uke.de (J.K.); tabea.hartung@kgu.de (T.I.H.); leopold.torster@uke.de (L.K.T.)

**Keywords:** immune checkpoint inhibitors, metastatic uveal melanoma, liver-directed therapy, cancer immunotherapy, embolization procedures, radiological procedures

## Abstract

**Simple Summary:**

Immune checkpoint inhibitors (ICI) have failed to overcome the high therapy resistance of Metastatic Uveal Melanoma (MUM) in contrast to the dramatic benefits of immunotherapy seen in many other tumor entities. Considering the poor clinical outcome and survival rates of MUM, the urgent need of new therapeutic approaches becomes apparent. This retrospective single-center cohort study of patients with liver-metastatic UM aims to investigate whether the combination of ICI and liver-directed therapies may improve the activity of immunotherapy against this highly malignant cancer. Our results demonstrate a response to MUM resistance to immunotherapy by combining liver-directed therapies and ICI, leading to improvement of overall survival (OS).

**Abstract:**

Uveal Melanoma (UM) is a rare disease; however, it is the most common primary intraocular malignant tumor in adults. Hematogenous metastasis, occurring in up to 50% of cases, mainly to the liver (90%), is associated with poor clinical course and treatment failure. In contrast to dramatic benefits of immunotherapy in many tumor entities, as seen in cutaneous melanoma, immune checkpoint inhibitors (ICI) do not achieve comparable results in Metastatic UM (MUM). The aim of this study was to investigate whether the combination of ICI with liver-directed therapies provides a potential survival benefit for those affected. This retrospective, single-center study, including *n* = 45 patients with MUM, compared the effect of combining ICI with liver-directed therapy (“Cohort 1”) with respect to standard therapies (“Cohort 2”) on overall survival (OS). Our results revealed a significant survival difference between Cohort 1 (median OS 22.5 months) and Cohort 2 (median OS 11.4 months), indicating that this combination may enhance the efficacy of immunotherapy and thus provide a survival benefit. There is an urgent need for randomized, prospective trials addressing the combination of liver-directed therapies and various strategies of immunotherapy (such as ICI; IMCgp100; personalized vaccines) in order to establish regimens which finally improve the prognosis of patients with MUM.

## 1. Introduction

Despite their common melanocytic origin, Uveal Melanoma (UM) differs significantly from cutaneous melanoma not only in terms of epidemiology, biology, clinical presentation, and metastasis, but also regarding treatment options, including their different response to immunotherapy [1,2].

As UM accounts for approximately five percent of all malignant melanomas, it is considered a rare disease. Nonetheless, it is the most common primary intraocular tumor in adults [3,4,5]. The global incidence of UM remains at 5.1 per 1,000,000 per year [6], showing a north–south gradient in Europe (8 per 1,000,000 in Norway and Denmark versus 2 per 1,000,000 in Spain and southern Italy) [7]. The average age at diagnosis is 58 years [8], while men are slightly more affected than women [7,9].

As the Uvea is the vascular layer of the eye, hematogenous metastasis occurs in up to 50% [3,4,10] and at an early stage [9], predominantly to the liver (89%) [10]. The metastatic stage is associated with poor prognosis [11]: 1-year survival ranges from 10–15% [12] to, regarding a recent meta-analysis, 43% [13], while median overall survival (OS) ranges from 4 to 15 months [4,11].

Several therapeutic options have been evaluated in the treatment of MUM. Surgical resection of liver metastases may provide longer-term survival, however, only isolated or oligo metastases and, thus, only a minor subgroup of those affected are amenable to surgery [9]. Systemic therapy regimens did not achieve significant benefits so far: the response rates to chemotherapy, adapting protocols from cutaneous melanoma [14], have been poor, ranging from 0 to 15% [2,15,16]. Moreover, neither targeted nor immunotherapies improved survival significantly [9]. Although UM-specific mutations in *GNAQ* and *GNA11* result in constitutive activation of MAPK- and PI3K-Akt-signaling [15,17,18], MUM did also not respond to MEK inhibitors (e.g., Selumetinib) in clinical trials [15,16]. Unfortunately, likewise, the high efficacy of ICI in cutaneous melanoma could not be transferred to MUM. FDA approval trials of Nivolumab and Ipilimumab [17,18,19] excluded patients with MUM [14,20] and clinical trials investigating their effect on MUM showed low response rates with no significant benefits in terms of PFS and OS [14,21,22,23].

In order to overcome these low response rates to systemic therapies [9], liver-directed procedures including transarterial chemoembolization (TACE), selective internal radiotherapy (SIRT), and immunoembolization have been established in the treatment of MUM, aiming to control the metastatic nature of UM while minimizing the systemic exposure to toxic agents [4]. Radiological embolization procedures combine local application of chemotherapeutic agents (e.g., Photomustine, Carmustine, and Cisplatin), radioactive isotopes (Yttrium-90, 90Y) [24], or glycoproteins such as Granulocyte-Macrophage Colony-Stimulating Factor (GM-CSF) [4], respectively, with arterial embolization (e.g., with Lipidol). Although there is only a limited number of prospective studies which address the efficacy of liver-directed therapies in MUM, a meta-analysis [13] demonstrated their superiority compared to other therapies (chemotherapy, immunotherapy, and targeted therapy) in terms of higher 6-month PFS (77% versus 26%) and 1-year OS (88% versus 42.5%) [13].

Survival rates of MUM have not been improved over years [6,25], and there is still a lack of standardized therapeutic regimens [26]. Consequently, combination strategies which intend to overcome this resistance are urgently needed. The combination of CTLA-4 and PD-1/PD-L1 inhibition was associated with a median OS of 16.1 months (95% CI, 12.9–19.3) [27]. The efficacy of dual ICI (Nivolumab plus Ipilimumab) as first-line treatment of MUM was evaluated in a multicenter phase II study by the *Spanish Multidisciplinary Melanoma Group* (GEM-1402) (2021) identifying a 1-year OS of 51.9% (95% CI, 38.3–65.5), with a median OS of 12.7 months [28]. Furthermore, the combination of systemic and liver-directed therapies was retrospectively evaluated showing a median OS of 17.8 months (95% CI, 16.6–19.4) versus 5.3 months (95% CI, 4.2–7.0) in the control group (systemic chemotherapy) [11]. A further improvement in survival (median OS 18.4 months) was achieved by combing liver-directed therapy and dual ICI (low-dose Ipilimumab and normal-dose Anti-PD-1) in 33 patients with metastatic melanoma. Of these, nine MUM patients, the vast majority (89%, *n* = 8) with liver metastases, were treated by liver-directed therapies before the initiation of dual ICI [29].

Taken together, the combination of liver-directed therapies and immunotherapy, in particular, may be promising in the treatment of MUM due to complementary effects [14]: Since liver-directed therapies lead to an increased release of tumor antigens and, thus, to a higher tumor mutational burden [30,31], the simultaneous use of immunotherapy could result in a greater immune response in MUM. Therefore, this retrospective study aims to investigate whether the additional use of liver-directed embolization procedures may overcome the resistance of MUM to immunotherapy and thus contribute to clinical benefit and prolonged survival. 

## 2. Materials and Methods

### 2.1. Study Sample

The present study is a retrospective, explorative analysis of all liver-metastatic UM patients who presented to the Department of Dermatology and Venereology of the University Medical Center Hamburg-Eppendorf between 2010 and April 2021. The further analyses included those patients whose medical history could adequately be assessed retrospectively (*n* = 45). Data were collected between January and April 2021 and documented anonymously. Data analysis was performed from 21 April 2021. 

**Figure 1:**
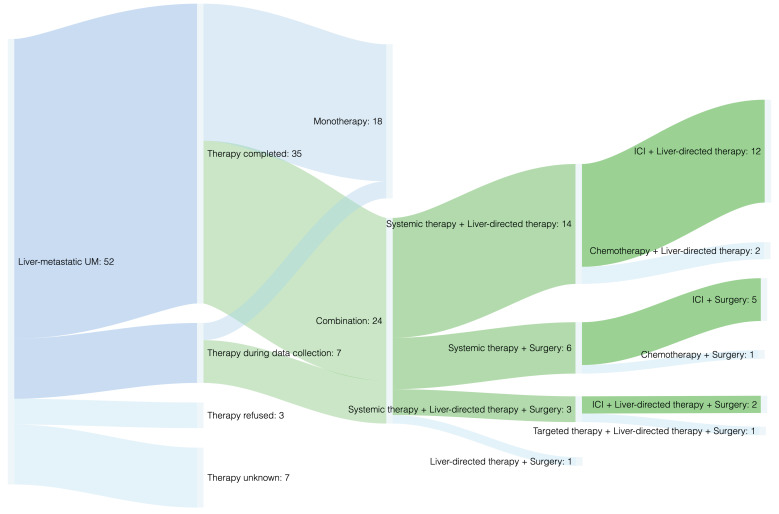
Therapy regimens. Note: the green path illustrates Cohort 1 including all patients treated with ICI plus liver-specific therapies (surgical resection of metastases and/or liver-directed therapies such as transarterial chemoembolization), which was compared to Cohort 2 (blue path, including any other therapy modalities, such as any monotherapies or any other combination therapies). For a detailed overview about the different therapies performed, see Table S1.

Figure 1 provides a structured overview of the cohort according to therapy procedures performed (see also Figure S1). Aiming to evaluate whether the efficacy of ICI may be enhanced by performing liver-specific therapies simultaneously; survival analysis was performed for two treatment groups: Cohort 1 (illustrated in green) and Cohort 2 (illustrated in blue) (Figure 1).

### 2.2. Statistical Analysis

The analysis’ primary endpoint was overall survival (OS) from diagnosis of liver metastasis to death. Kaplan–Meier estimates were used to evaluate differences in median OS between groups. The main comparisons referred to two cohorts subdivided by therapy regimens: Cohort 1 included all patients who were treated with ICI plus liver-specific therapy (liver-directed therapy and/or surgical resection), and Cohort 2 included all patients who were treated differently (any monotherapy; other systemic therapies expect ICI in combination with liver-specific therapies; any liver-specific therapies without systemic therapies) (Table S1). All patient cases were presented to the multidisciplinary tumor board and a treatment recommendation was made. The therapy decisions were made in the context of a participatory decision between patient and supervising physician.

In addition, the effects of other clinical parameters on survival were assessed exploratively, e.g., sex, age at diagnosis of liver metastasis, presence of other metastasis, blood levels of LDH, S100, Eosinophile Granulocytes, and Neutrophil-to-Lymphocyte Ratio (NLR). The differences in survival were evaluated using the log rank test with *p* ≤ 0.05 considered as statistically significant. Cox proportional hazards models were used to evaluate the effects of clinical variables on survival. All analyses were performed using Statistical Package for the Social Sciences (IBM SPSS Statistics version 25, IBM Corporation, Stuttgart, Germany). 

## 3. Results

### 3.1. Cohort Characteristics

Data were retrospectively collected from 52 patients with liver-metastatic UM. Among them, seven cases were excluded from further analysis due to a lack of information. Thus, *n* = 45 cases were selected for further analysis. The majority was male (73.3%, *n* = 33), predominantly affected by choroidal melanoma (84.4%, *n* = 38). The median age was 59 years (range 21–79) at initial diagnosis and 63 years (range 21–79) at diagnosis of liver metastasis. Median time from initial diagnosis to liver metastases was 1.7 years (range 0–16.4). Liver-metastases, defined as inclusion criterion (100%, *n* = 45), were treated in 93.3% (*n* = 42). Extrahepatic distant metastases were reported in 64.4% of cases (*n* = 29), including lung (53.3%, *n* = 24), bone (26.7%, *n* = 12), lymph node (22.2%, *n* = 10), cutaneous/subcutaneous (22.2%, *n* = 10), and, less frequently, brain (8.9%, *n* = 4) metastases (Table 1). LDH at baseline was elevated in 68.6%, S100 in 25.9%, and Eosinophile Granulocytes in 12.5%. NLR ≥ 3.5 was seen in 43.8% (Table S2). In total, 71.1% (*n* = 32) of patients were deceased and 22.2% (*n* = 10) alive, of whom seven patients (15.6%) were currently under therapy. For three (6.7%) palliative cases no date of death could be determined retrospectively (Table 1).

### 3.2. Therapy of Metastatic Uveal Melanoma

Therapy regimens of *n* = 42 patients with liver-metastatic UM were evaluated (Figure 1 and Figure S1, Table S1). Due to the lack of consensus guidelines, as well as the development of new immunotherapeutic strategies, patients between 2010 and 2021 were treated heterogeneously (Table S1). During data collection, treatment had been completed in 35 cases (83.3%). Therapy regimens included systemic therapy in 83.3% (*n* = 35), surgical liver resection in 26.2% (*n* = 11), and liver-directed therapy in 54.8% (*n* = 23), either performed as mono- (42.9%, *n* = 18) or as combined therapy (57.1%, *n* = 24) (Figure 1, Table S1). Predominantly, the combination of systemic and liver-directed therapies was performed (33.3%, *n* = 14), most frequently dual ICI together with TACE (19.0%, *n* = 8) (Table S1).

### 3.3. Evaluating a Potential Survival Benefit by Combining ICI with Liver-Directed Therapies

**Figure 2:**
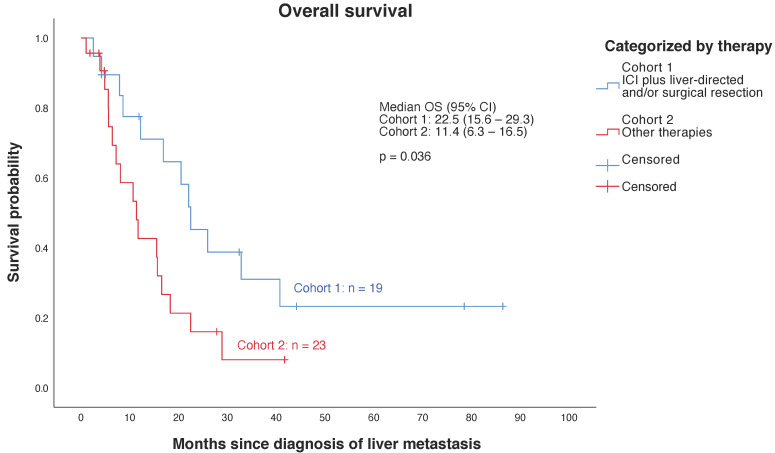
Kaplan–Meier estimates for overall survival (OS) from liver metastasis to death according to therapy. Note: Cohort 1 includes all patients treated with ICI plus liver-specific therapies (surgical resection of liver metastases and/or liver-directed therapies), which was compared to Cohort 2 including any other therapy regimens such as any monotherapies and any other combination regimens (Table S1).

First, a descriptive survival analysis was performed for the entire cohort of *n* = 45 patients showing a median OS of 15.50 months (95% CI, 8.59–22.41). Further analyses excluded patients whose metastatic disease was not treated (*n* = 3). Patients whose treatment was not completed during data collection were considered in the statistical analysis, presented in Figure 2; further analyses focused on the comparison between the two therapy cohorts (Figure 1, Table S1). Survival was significantly higher (*p* = 0.036) in Cohort 1 showing a median OS of 22.47 months (95% CI, 15.62–29.32) compared to a median OS of 11.37 months (95% CI, 6.26–16.48) in Cohort 2 (Figure 2, Table S1).

**Figure 3:**
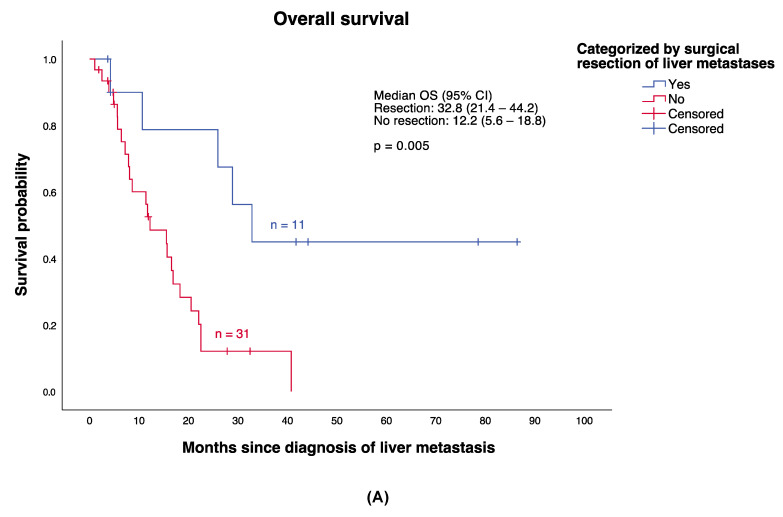

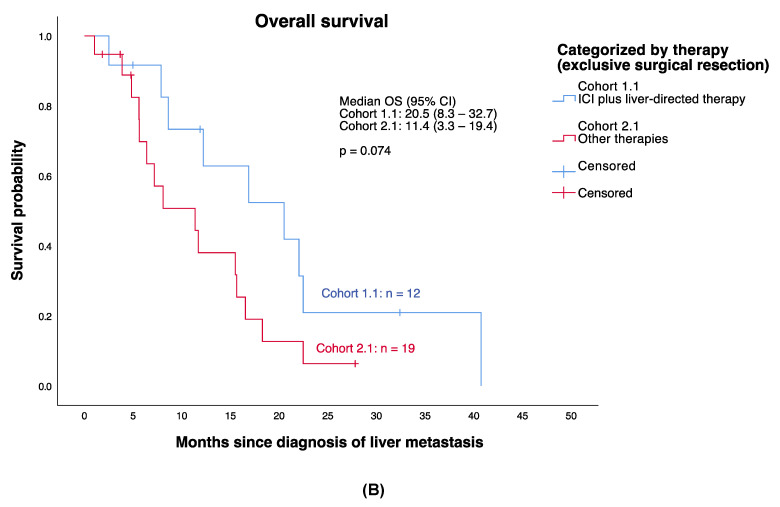
Kaplan–Meier estimates for overall survival (OS) from liver metastasis to death according to therapy, considering the impact of surgical resection of liver metastases. (**A**) Surgical resection of liver metastases indicated a highly significant impact on OS of patients with MUM. (**B**) In order to avoid corresponding bias, comparison of survival between Cohort 1 and Cohort 2 were additionally performed only for those whose metastases were not surgically resected (*n* = 31), referred to as Cohort 1.1 (ICI plus liver-directed therapy exclusive surgical treatment) and Cohort 1.2 (monotherapies, combinations of systemic therapy and liver-directed therapies excluding surgical treatment).

A categorization by surgical resection of liver metastases demonstrated a highly significant difference (*p* = 0.005) in OS between those patients who underwent surgical resection (median OS 32.8 months; 95% CI, 21.42–44.18; *n* = 11) and those who did not (median OS 12.20 months; 95% CI; 5.58–18.82, *n* = 31) (Figure 3A). Consequently, the comparison between treatment Cohort 1 and Cohort 2 were additionally performed only for those whose liver metastases were not resected (*n* = 31), thus avoiding corresponding bias (referred to as Cohort 1.1 and Cohort 2.1, (Figure 3B). Accordingly, Cohort 1.1 included all patients who received ICI in combination with liver-directed therapies (*n* = 12) and Cohort 2.1 all patients who received monotherapies or other combinations of systemic and liver-directed therapies (*n* = 19) exclusive surgical resection. Median OS was 20.50 months (95% CI, 8.34–32.66) in Cohort 1.1 and 11.37 months (95% CI, 3.29–19.44) in Cohort 2.1. Accordingly, a trend towards a higher median OS in Cohort 1.1 could be seen, even though this difference did not reach statistical significance (*p* = 0.074) (Figure 3B).

### 3.4. Identification of Clinical Variables Associated with Survival

In addition, univariate and multivariate Cox regressions were performed (Table 2) to examine the simultaneous influence of independent variables on survival. For this purpose, corresponding continuous variables were dichotomized. The significant superiority in terms of OS for the combination of ICI and liver-specific therapies (referred to above as Cohort 1) could be confirmed (HR = 2.2, *p* = 0.041). Differences in median OS were also significant for the variables of age at diagnosis of liver metastases (cut-off: 55 years, *p* = 0.043) and for the laboratory parameters LDH (cut off: 246 U/L, *p* = 0.030), S100 (cut-off: 0.15 µg/L, *p* = 0.013) and NLR (cut-off: 3.5, *p* = 0.005) (Figures S2–S4). Moreover, patients with additional lung metastases had a significant lower risk to reach the endpoint than those without lung metastases (HR = 0.47, *p* = 0.044). Median OS with lung metastasis was 20.50 months (95% CI, 13.15–27.85) and without lung metastasis was 8.07 months (95% CI, 2.60–13.53) (Figure S5).

Higher serum levels of NLR (<3.5 versus ≥3.5: HR = 4.75, *p* = 0.048), S100 (<0.15 µg/L versus ≥0.15 µg/L: HR = 3.99, *p* = 0.048) and LDH (<246 U/L versus ≥246 U/L: HR = 7.41, *p* = 0.071) indicated a higher probability to reach the defined endpoint (Table 2, multivariate analysis, *p* = 0.002). However, LDH did not reach the criterion for statistical significance of *p* ≤ 0.05 in this model. Furthermore, the influence of age at diagnosis of liver metastasis (<55 years versus ≥55 years: HR = 2.67, *p* = 0.034) and the presence of lung metastasis (no versus yes: HR = 0.45, *p* = 0.033) on OS could be confirmed. Table 2 provides a detailed overview of the Cox regression analyses including Hazard Ratios, 95% Confidence Intervals and *p*-values.

## 4. Discussion

Despite excellent local disease control, UM metastasizes frequently and early to the liver [10], where response rates to any therapeutic options and, thus, prognosis is poor. ICI currently represents the most effective treatment available for MUM apart from clinical trials [27], although results are not comparable to those in cutaneous melanoma [9,15,27,31,32]. As MUM is considered to be a *cold* tumor [27] and is characterized by *a low tumor mutational burden* [27,30], its resistance to therapy can be explained by being unrecognizable for the immune system. Furthermore, it is of significant relevance that the liver has acquired specific mechanisms of immune tolerance, such as high secretion of anti-inflammatory mediators (IL-10, TGF-ß) and low levels of cytotoxic T-cells [33]. The so-called *liver tolerance effect* [34] causes an immunosuppressive niche in which MUM remains protected from the immune system and, thus, from ICI. Understanding these mechanisms of therapy resistance [35], the need for complementary or synergistic treatment regimens is crucial. In particular, response rates could be improved by combing ICI and liver-directed therapies, as local embolization leads to increased release of tumor antigens which could induce a stronger response to the simultaneous use of systemic ICI [14].

To date, only a few trials have investigated the effect of combined therapies on survival of patients with MUM. For instance, two clinical trials evaluated the efficacy of dual ICI (Nivolumab plus Ipilimumab) showing a median OS of 16.1 months (95% CI, 12.9–19.3) [27] and 12.7 months [28], respectively. The combination of systemic chemotherapy and liver-directed therapy led to a median OS of 17.8 months (95% CI, 16.6–19.4) [11]. However, only one retrospective trial [29] evaluated the combination of dual ICI (low-dose Ipilimumab plus Nivolumab) and liver-directed therapy in eight UM patients with liver metastases showing a median OS of 18.4 months [29]. In the context of liver-directed radiotherapy in combination with ICI, the concepts of peri-induction radiotherapy (PIR) and post-escape radiotherapy (PER) are distinguished.

Our retrospective trial goes beyond previous analyses by demonstrating the importance of the combination of ICI (Nivolumab plus Ipilimumab or Nivolumab/Ipilimumab alone) and liver-specific therapies (surgical resection and/or liver-directed therapies) providing a significant survival benefit (*p* = 0.036) for MUM patients. Median OS was 22.47 months in Cohort 1 versus 11.37 months in Cohort 2 (Figure 2). In comparison to previous analyses described above, these results may indicate that median OS can be significantly improved by combining ICI and liver-specific procedures. The major impact of surgical resection on survival of patients showing liver metastases must be taken into account [9]. Despite its crucial impact (*p* = 0.005) on survival, surgical resection is limited to patients with single or few liver metastases, resulting in a highly selected collective of patients amenable to surgical resection. Thus, bias must be considered by interpreting the shown analysis [14,36]. To prevent such potential bias in the treatment group comparisons, survival analyses were performed on patients who underwent surgical resection and those who did not, respectively (Figure 3B). Altogether, survival analyses indicate that there may be a survival benefit in case of ICI and liver-directed therapy being combined, even though the results did not reach statistical significance (*p* = 0.074).

Moreover, treatment resistance of MUM can also be addressed by novel immune-based approaches [32,35,37,38] aiming to extend the efficacy of immunotherapy to immunological *cold* tumors. For instance, IMCgp100 (Tebentafusp) represents a bispecific fusion protein with both targeting and effector elements. The targeting domain constitutes a soluble T-cell receptor (TCR) that recognizes the melanoma-associated antigen gp100 presented by HLA-A*02-01 that is expressed in approximately 50% of patients with UM. The effector domain includes an anti-CD3 single-chain variable fragment (scFv) which redirects T-cells to kill gp100 expressing UM cells [35,39]. The recent study IMCgp100–202 [ClinicalTrials.gov, identification number: NCT03070392] compares safety and efficacy of IMCgp100 versus standard (investigator’s choice) therapies as first-line treatment of patients with HLA-A*02-01^+^ MUM. Significant improvement in OS with respect to treatment with Tebentafusp (median OS 21.7 months) leads to its approval being expected sometime between the end of 2021 to early 2022 [40]. The combination of Tebentafusp and ICI in sequence will also be evaluated in further trials.

A prognostic score for patients with MUM, including the variables time to metastatic diagnosis, presence of bone metastases, and LDH, was recently developed [41,42,43]. Based on the results of our analysis, the parameters age (cut-off: ≥55 years), as well as LDH, S100, and NLR (cut-off: 3.5), should be considered in case of prognosis assessment. Our analysis highlights that the presence of additional lung metastases in case of liver-metastatic UM leads to better survival, underscoring the hypothesis that extrahepatic distant metastases might be more accessible to ICI. Immunotherapy, thus, results in a higher induction of immune cells against corresponding tumor cells metastasized to the liver [39,44].

The strengths of the study included the relatively large sample size of *n* = 45 patients treated for liver metastases at the University Medical Center Hamburg-Eppendorf between 2010 and 2021, considering the low incidence of UM. To our knowledge, this is the first study which compared ICI plus liver-directed therapies with other therapy modalities. Furthermore, various factors influencing the survival of patients with MUM were considered in our analysis. Heterogeneous therapy regimens enabled a comparison of different treatment strategies.

Nevertheless, there are limitations of this study. Division of the study population into only two cohorts was necessary due to the cohort size. Thus, a distinction between surgical, radiotherapeutic, and interventional drug liver-directed therapy was not possible in the context of this study. Furthermore, the retrospective study design could not avoid incomplete clinical data, especially the long follow-up period as an obstacle to retrospective data collection. Analyzing the clinical outcome of patients treated in a single academic center might bias generalization to a broader community. Moreover, a thorough genetic analysis including SF3B1 driver mutations [45] of our study cohort has not been performed. The continuous development of therapeutic options, including newly approved regimens in the field of ICI, as well as in the field of liver-specific therapy in the recruitment period 2010 to 2021, also represents a limitation.

## 5. Conclusions

As a result, we demonstrated a survival benefit for patients with MUM treated with both liver-specific therapies and ICI. Our analyses indicate the possibility of overcoming the resistance of MUM representing a highly malignant cancer with low tumor mutational burden. These combinations of therapy strategies and inclusion of novel immunotherapeutic approaches are urgently needed to be evaluated by prospective clinical studies to improve the efficacy of anticancer immunotherapy and, thus, the survival of patients with MUM.

## Figures and Tables

**Table 1:** Cohort characteristics.

Characteristics	No. (%)	Cumulated %	Median Years (Range)
**Sex**			
Male	33 (73.3)	73.3	
Female	12 (26.7)	100.0	
**Tumor location**			
Choroidal Melanoma	38 (84.4)	84.4	
Ciliary body Melanoma	7 (15.6)	100.0	
**Age at diagnosis**			
Primary UM			59 (21–79)
Liver metastasis			63 (21–79)
Time from primary diagnosis to liver metastasis			1.7 (0.0–16.4)
**Distant metastasis**			
Liver	45 (100.0)		
Extrahepatic metastasis	29 (64.4)		
Lung	24 (53.3)		
Bone	12 (26.7)		
Lymph node	10 (22.2)		
Skin/Subcutaneous	10 (22.2)		
Brain	4 (8.9)		
Other	3 (6.7)		
**UM specific mortality**			
No	10 (22.2)	22.2	
Yes	32 (71.1)	93.3	
Unknown	3 (6.7)	100.0	

Note: the descriptive statistics included patients with liver-metastatic UM whose history of disease and therapy could be retrospectively reconstructed (*n* = 45).

**Table 2:** Cox regression analysis (univariate analysis and multivariate analysis).

Characteristics	N ^1^	Hazard Ratio	95% CI	*p*-Value
Univariate analysis				
**Demographic variables**				
Age at diagnosis liver metastases (<55 vs. ≥55 years)	42	2.553	1.03–6.32	0.043
Sex (male vs. female)	42	0.642	0.28–1.46	0.290
Lung metastases (no vs. yes)	42	0.468	0.22–0.98	0.044
**Laboratory values**				
LDH (<246 U/L vs. ≥246 U/L)	33	2.878	1.11–7.47	0.030
S100 (<0.15 µg/L vs. ≥0.15 µg/L)	26	4.638	1.37–15.65	0.013
NLR (<3.5 vs. ≥3.5)	31	4.738	1.59–14.14	0.005
**Therapy of metastastic disease**				
Surgical resection of liver metastases (no vs. yes)	42	0.247	0.0–0.69	0.008
ICI plus liver-directed therapy and/or surgical resection vs. other treatment	42	2.204	1.03–4.70	0.041
Multivariate analysis				
**(1) Multivariate Cox-regression**	22			0.002
NLR (<3.5 vs. ≥3.5)		4.746	1.01–22.22	0.048
S100 (<0.15 µg/L vs. ≥0.15 µg/L)		3.994	1.01–15.77	0.048
LDH (<246 U/L vs. ≥246 U/L)		7.411	0.85–64.96	0.071
**(2) Multivariate Cox-regression**	42			0.010
Age at diagnosis liver metastasis (<55 vs. ≥55 Jahre)		2.665	1.08–6.60	0.034
Lung metastasis (no vs. yes)		0.447	0.21–0.94	0.033

Note: ^1^ cases available for analysis.

## Data Availability

The datasets used and/or analyzed during the current study are available from the corresponding author on reasonable request.

## Supplementary Materials

The following are available online at www.mdpi.com/article/10.3390/cancers13246390/s1: Table S1: Therapy of liver-metastatic Uveal Melanoma; Table S2: Laboratory values at Baseline; Figure S1: Flow chart on cohort composition, Figure S2: Effect of LDH at Baseline on overall survival of patients with liver-metastatic Uveal Melanoma; Figure S3: Effect of S100 at Baseline on overall survival of patients with liver-metastatic Uveal Melanoma; Figure S4: Effect of NLR at Baseline on overall survival of patients with liver-metastatic Uveal Melanoma; Figure S5: Effect of lung metastases on overall survival of patients with liver-metastatic Uveal Melanoma.
