# Supplementary material for: Combination of Immune Checkpoint Inhibitors and Liver-Specific Therapies in Liver-Metastatic Uveal Melanoma: Can We Thus Overcome Its High Resistance?
Source: Cancers (Basel). 2021 Dec 20;13(24):6390. doi: 10.3390/cancers13246390 (PMC8699813; doi:10.3390/cancers13246390)
Supplement: Supplementary file 1 [file cancers-13-06390-s001.zip › cancers-1468362-supplymentary.pdf]

cancers

Article

Combination of immune checkpoint inhibitors and liver-specific therapies in liver-metastatic uveal melanoma: Can we thus overcome its high resistance?

Special issue: Advances in the Treatment of Metastatic Uveal Melanoma

SUPPLEMENTARY MATERIALS

**Table S1**	Therapy of liver-metastatic Uveal Melanoma
**Table S2**	Laboratory values at Baseline
**Figure S1**	Flow chart on cohort composition
**Figure S2**	Effect of LDH at Baseline on overall survival of patients with liver-metastatic Uveal Melanoma
**Figure S3**	Effect of S100 at Baseline on overall survival of patients with liver-metastatic Uveal Melanoma
**Figure S4**	Effect of NLR at Baseline on overall survival of patients with liver-metastatic Uveal Melanoma
**Figure S5**	Effect of lung metastases on overall survival of patients with liver-metastatic Uveal Melanoma

Table S1. Therapy of liver-metastatic Uveal Melanoma

Therapy of liver-metastatic UM	n (%)	Cumulated %
**Therapy completed**		
No	3 (6.7)	6.7
Yes	35 (77.8)	84.4
Therapy during data collection	7 (15.6)	100.0
**Therapies performed**		
Surgical resection of liver metastases	11 (26.2)	
Liver-directed therapy	23 (54.8)	
Systemic therapy	35 (83.3)	
**Liver-directed therapy**		
TACE	20 (44.4)	
SIRT	2 (4.4)	
Chemosaturation	1 (2.2)	
**Systemic therapy**		
Dual ICI (Ipilimumab+Nivolumab)	20 (44.4)	
Anti-PD-1 (Nivolumab/Pembrolizumab)	4 (8.9)	
Chemotherapy (Gemcitabine/Decarbazine)	6 (13.3)	
Anti-CTLA-4 (Ipilimumab)	5 (11.1)	
Targeted Therapy (Trametinib)	3 (6.7)	

Therapy regimens	n (%)[1]	Cumulated %	Median OS (months)	n events[2]
Cohort 1	**19 (45.2)**			
Dual ICI + Surgical resection	4 (9.5)	52.4	55.65	1
Anti-PD-1 + Surgical resection	1 (2.4)	54.8	4.2	1
Dual ICI + TACE	8 (19.0)	76.2	10.23	6
Dual ICI + Chemosaturation	1 (2.4)	78.6	32.4	0
Anti-PD-1 + SIRT	1 (2.4)	81.0	22.5	1
Anti-CTLA-4 + TACE	2 (4.8)	85.8	18.68	2
Dual ICI + TACE + Surgical resection	1 (2.4)	93.0	25.93	1
Anti-PD-1 + SIRT + Surgical resection	1 (2.4)	95.4	44.13	0
Cohort 2	**23 (54.8)**			
Surgical resection alone	1 (2.4)	2.4	42	0
Liver-directed therapy (TACE) alone	5 (11.9)	14.3	7.17	5
Dual ICI alone	6 (14.3)	28.6	4.25	4
Anti-PD-1 alone	1 (2.4)	31.0	5.63	1
Chemotherapy + Anti-CTLA-4	3 (7.1)	38.1	15.63	3
Targeted therapy alone	1 (2.4)	40.4	11.37	1
IMCgp-100 alone	1 (2.2)	42.9	4.77	0
Chemotherapy + Surgical resection	1 (2.4)	57.2	10.7	1
Chemotherapy + TACE	2 (4.8)	90.6	25.13	1
Targeted therapy + TACE + Surgical resection	1 (2.4)	97.7	28.87	1
TACE + Surgical resection	1 (2.4)	100.0	3.67	0

Note. [1]Percentages refer to n=patients who underwent treatment. [2]events are defined as death, all other patients were censored.

Table S2. Laboratory values at Baseline

**Laboratory: Baseline**[1]	**No. (%)**[2]	**Median**	**Interquartile difference**	**Elevated, no. (%**[4]**)**
LDH (120-246 U/l)	35 (77.8)	293	214	24 (68.6)
S100 (-0.15 µg/l)	27 (60.0)	.06	.13	7 (25.9)
Eosinophile Granulocytes (0.1-0.5 Mrd/l)	32 (71.1)	.17	.23	4 (12.5)
NLR[3] (-3.5)	32 (71.1)	3.12	2.2	14 (43.8)

Note. [1]"*Baseline*": Laboratory values prior to initial system therapy. If no systemic therapy was given, an alternative laboratory was chosen. [2] Since not all laboratory values were available for every patient, "*No. (%)*" indicates how many laboratory values were included in the statistics. [3] Neutrophil-to-Lymphocyte-Ratio. [4] Elevated parameters in relation to the total number of parameters collected (= valid percentages).

Figure S1

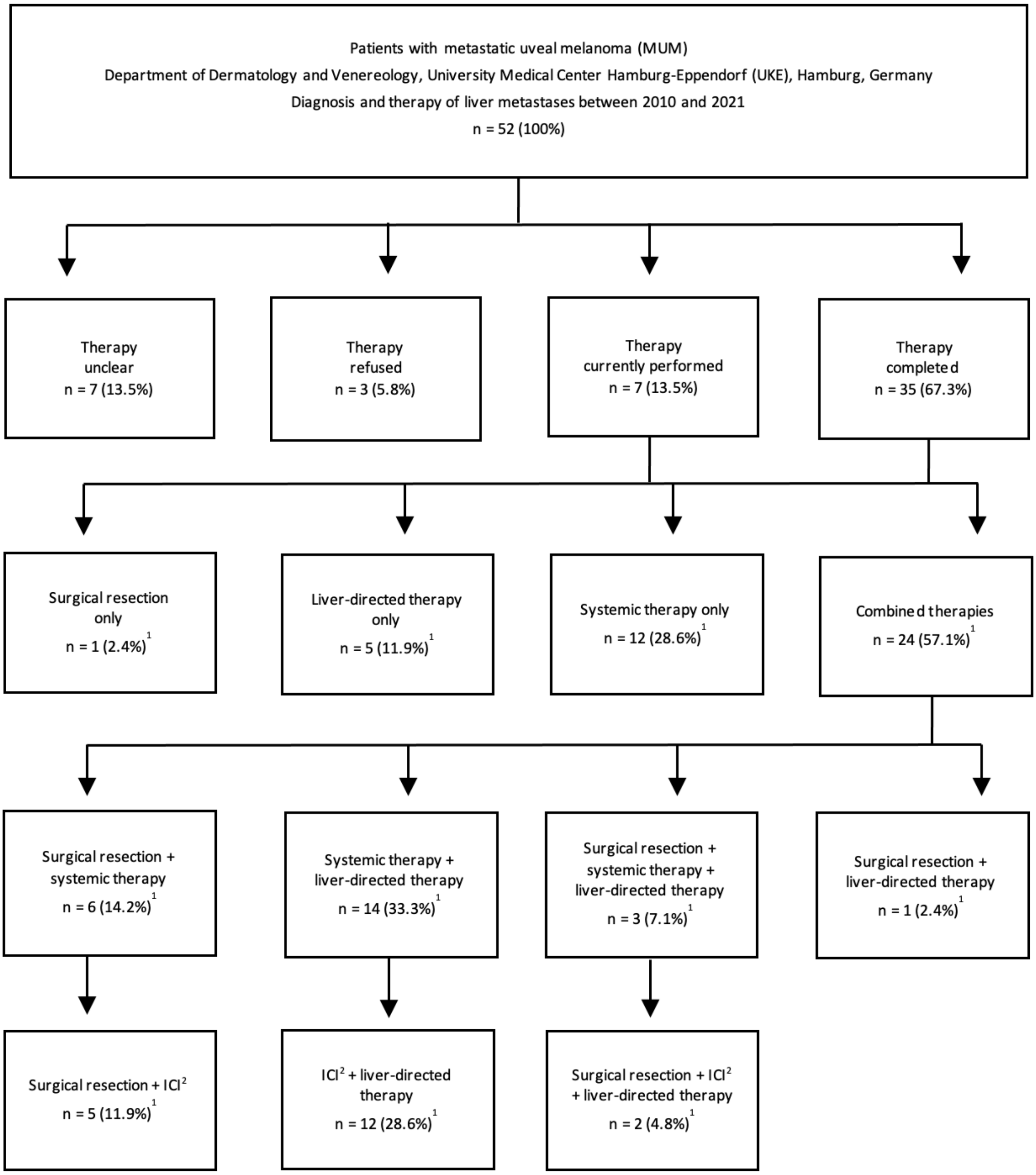

Figure S1 Flow chart on cohort composition. *Note.* [1]Percentages refer to a total of n=42 patients whose metastatic disease has been treated. [2]"ICI" includes dual anti-PD-1 (Nivolumab) plus anti-CTLA-4 (Ipilimumab) as well as anti-PD-1 (Nivolumab or Pembrolizumab) or anti-CTLA-4 (Ipilimumab) alone.

Figure S2

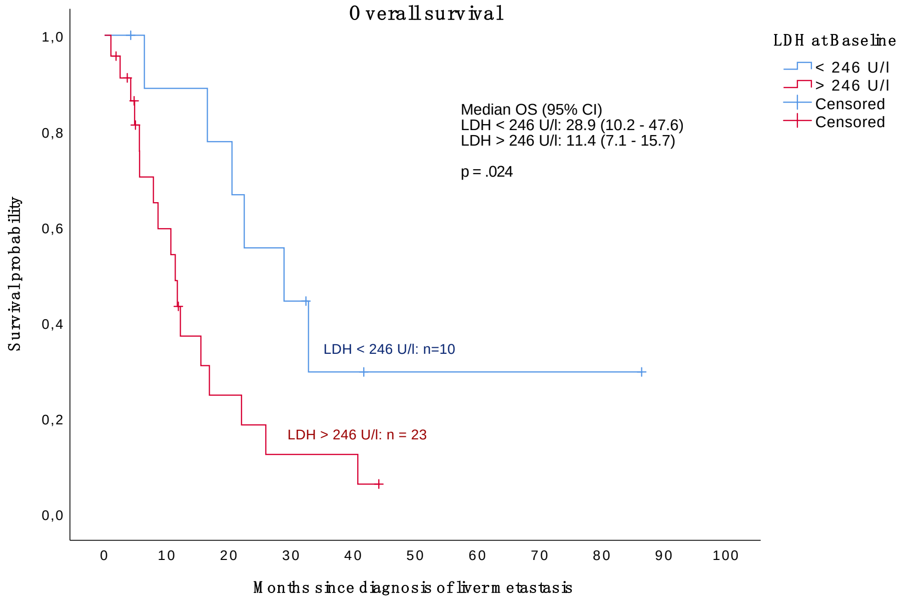

Figure S2 Effect of *LDH* at Baseline on overall survival of patients with liver-metastatic Uveal Melanoma (n=33; Cut-Off: 246 U/l).

Figure S3

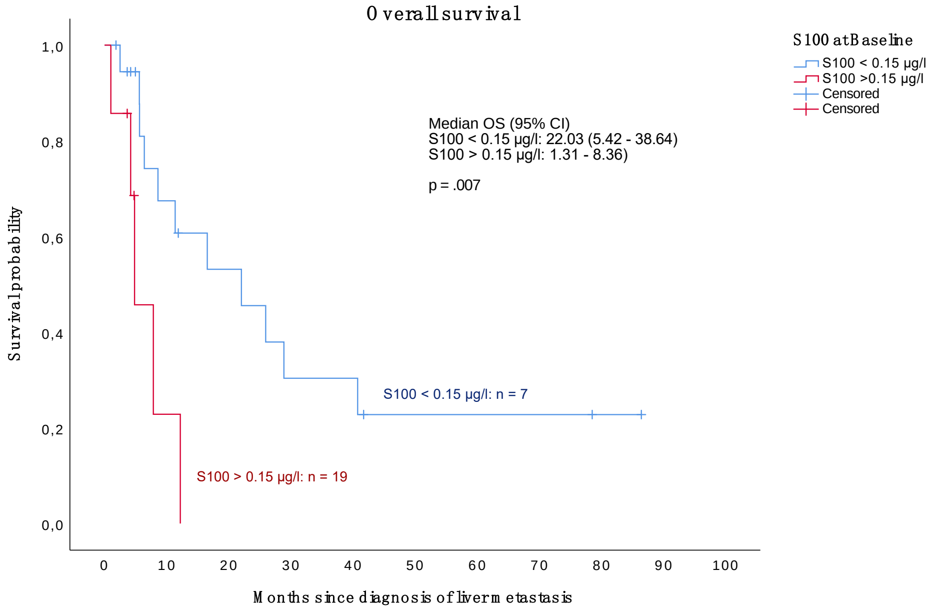

Figure S3 Effect of *S100* at Baseline on overall survival of patients with liver-metastatic Uveal Melanoma (n=26; Cut-Off: 0.15 µg/l).

Figure S4.

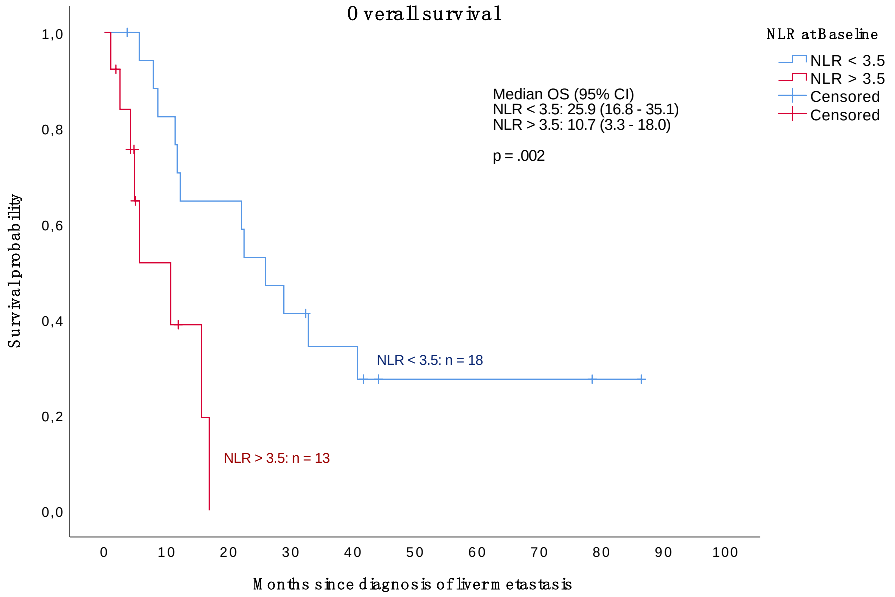

Figure S4 Effect of *NLR* (Neutrophil-to-Lymphocyte Ratio) at Baseline on overall survival of patients with liver-metastatic Uveal Melanoma (n=31; Cut-Off: 3.5).

Figure S5.

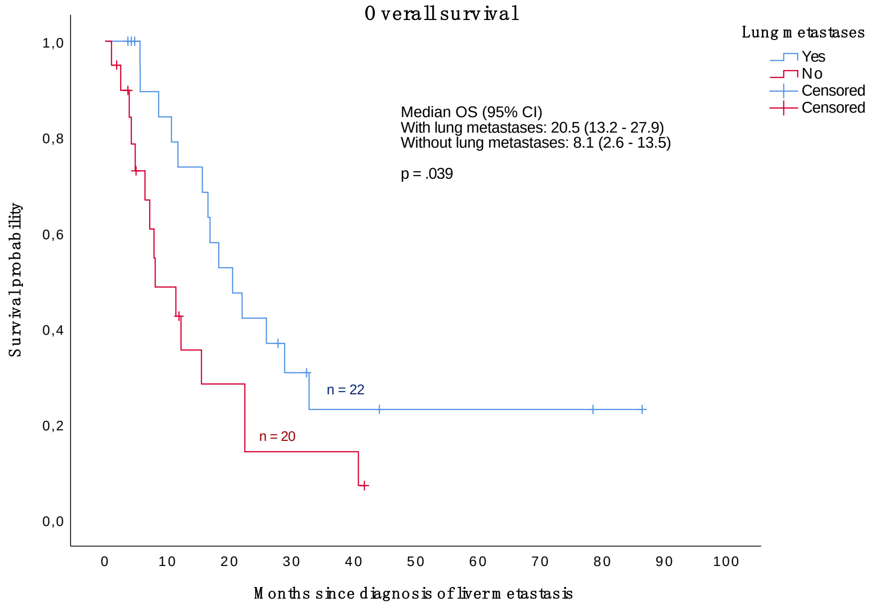

Figure S5 Effect of lung metastases on overall survival of patients with liver-metastatic Uveal Melanoma (n=42).
